# *In vitro* and *in vivo* optimized reconstruction for low-keV virtual monoenergetic photon-counting detector CT angiography of lower legs

**DOI:** 10.1186/s41747-024-00481-x

**Published:** 2024-08-01

**Authors:** Dirk Graafen, Willi Bart, Moritz C. Halfmann, Lukas Müller, Lukas Hobohm, Yang Yang, Achim Neufang, Christine Espinola-Klein, Michael B. Pitton, Roman Kloeckner, Akos Varga-Szemes, Tilman Emrich

**Affiliations:** 1 Department of Diagnostic and Interventional Radiology, University Medical Center of the Johannes Gutenberg-University Mainz, Mainz, Germany; 2 German Center for Cardiovascular Research (DZHK), Partner-Site Rhine-Main, Mainz, Germany; 3 Department of Cardiology, University Medical Center of the Johannes Gutenberg-University Mainz, Mainz, Germany; 4 Center for Thrombosis and Hemostasis (CTH), University Medical Center of the Johannes Gutenberg-University Mainz, Mainz, Germany; 5 Department of Cardiac and Vascular Surgery, University Medical Center of the Johannes Gutenberg-University Mainz, Mainz, Germany; 6 Institute of Interventional Radiology, University Hospital Schleswig-Holstein, Campus Lübeck, Lübeck, Germany; 7 Division of Cardiovascular Imaging, Department of Radiology and Radiological Science, Medical University of South Carolina, Charleston, SC USA

**Keywords:** Below-the-knee, CT angiography, Photon-counting detector CT, Quantum iterative reconstruction, Reconstruction kernel

## Abstract

**Background:**

Lower extremity peripheral artery disease frequently presents with calcifications which reduces the accuracy of computed tomography (CT) angiography, especially below-the-knee. Photon-counting detector (PCD)-CT offers improved spatial resolution and less calcium blooming. We aimed to identify the optimal reconstruction parameters for PCD-CT angiography of the lower legs.

**Methods:**

Tubes with different diameters (1–5 mm) were filled with different iodine concentrations and scanned in a water container. Images were reconstructed with 0.4 mm isotropic resolution using a quantitative kernel at all available sharpness levels (Qr36 to Qr76) and using different levels of quantum iterative reconstruction (QIR-2–4). Noise and image sharpness were determined for all reconstructions. Additionally, CT angiograms of 20 patients, reconstructed with a medium (Qr44), sharp (Qr60), and ultrasharp (Qr72) kernel at QIR-2-4, were evaluated by three readers assessing noise, delineation of plaques and vessel walls, and overall quality.

**Results:**

In the phantom study, increased kernel sharpness led to higher image noise (*e.g.*, 16, 38, 77 HU for Qr44, Qr60, Qr72, and QIR-3). Image sharpness increased with increasing kernel sharpness, reaching a plateau at the medium-high level 60. Higher QIR levels decreased image noise (*e.g.*, 51, 38, 25 HU at QIR-2–4 and Qr60) without reducing vessel sharpness. The qualitative *in vivo* results confirmed these findings: the sharp kernel (Qr60) with the highest QIR yielded the best overall quality.

**Conclusion:**

The combination of a sharpness level optimized reconstruction kernel (Qr60) and the highest QIR level yield the best image quality for PCD-CT angiography of the lower legs when reconstructed at 0.4-mm resolution.

**Relevance statement:**

Using high-resolution PCD-CT angiography with optimized reconstruction parameters might improve diagnostic accuracy and confidence in peripheral artery disease of the lower legs.

**Key Points:**

- Effective exploitation of the potential of PCD-CT angiography requires optimized reconstruction parameters.
- Too soft or too sharp reconstruction kernels reduce image quality.
- The highest level of quantum iterative reconstruction provides the best image quality.

**Graphical Abstract:**

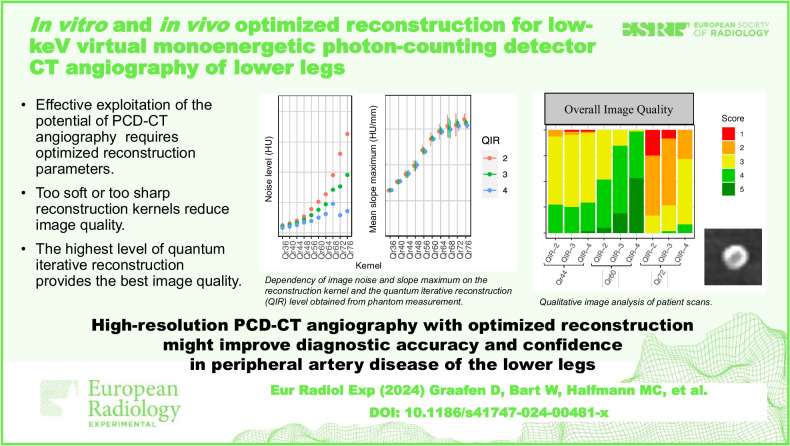

## Background

Lower extremity computed tomography (CT) angiography is a widely adapted medical imaging technique that enables the comprehensive evaluation of the peripheral vessels in individuals with peripheral artery disease, aneurysms, and trauma-related complications, and is used for both pre-interventional evaluation and postinterventional follow-up [1–3]. Previous studies reported a comparable diagnostic performance of CTA to digital subtraction angiography (DSA) [4, 5]. However, CTA diagnostic performance for the below-the-knee (BTK) arteries is typically limited due to their smaller vessel diameter, especially in combination with extensive arterial wall calcifications [6]. Despite those various developments in computed tomography (CT) technology, such as small field-of-view reconstructions [7], virtual monoenergetic image (VMI) reconstruction, and dual-energy CT-based calcium removal [8–10], have demonstrated improvements in imaging of the BTK arteries, the diagnostic accuracy of CTA remains inferior to that of DSA.

A new generation of CT technology, based on a photon-counting detector (PCD), has recently received clinical approval. These PCDs are made of semiconductor crystals and have the unique capability of directly converting x-ray photons into electrical signals, allowing the detection and measurement of individual photons and their respective energies [11–13]. The PCD-CT technology offers several advantages over conventional energy-integrating detector (EID) CT, including improved dose efficiency, reduced image noise, and enhanced contrast-to-noise ratios [14–20]. Since the conversion process into optical photons is eliminated, PCDs do not require reflecting lamellae, therefore enabling improved spatial resolutions down to the submillimeter range. Additionally, PCDs enable inherently to capture spectral data. These advantages hold the potential to further improve BTK CTA.

While recent studies investigated the benefits of coronary PCD-CTA [21–25], studies on the lower extremity remain sparse. Two previous studies demonstrated improvements by PCD-CT over EID-CT for lower extremity CTA [26, 27] in which the impact of different keV levels of the VMIs or different radiation dose levels was investigated. Indeed, image quality further depends on additional parameters such as the reconstruction kernel, especially its sharpness level, and the strength level of the applied iterative reconstruction algorithm, which has not been investigated for run-off PCD-CTA so far. We hypothesized that exploiting the potential of PCD-CT to improve the image quality of run-off CTA crucially depends on the combination of these reconstruction parameters.

Therefore, the aim of this study was to identify the optimal reconstruction settings for PCD-CTA of the lower legs both in phantoms and patients.

## Methods

### Phantom study

#### Experimental setup

Two 18.5 × 12 × 12 cm plastic containers, filled to approximately 60% with water, were positioned side-by-side on the CT table to simulate the two lower legs. Silicone tubes with five different inner diameters (1, 2, 3, 4, and 5 mm) and a wall thickness of 1 mm were placed sequentially in the center of one of the water containers. To simulate different contrast agent concentrations, a serial dilution of an iodine contrast medium with an initial concentration of 370 mg I/mL (Ultravist 370, Bayer Vital, Leverkusen, Germany) was prepared. First, the contrast agent was diluted with saline by a factor of 1:10, which was the highest iodine concentration used for the experiments. Three additional steps with a dilution factor of 1:2 were performed. Together with a pure saline probe, this resulted in five different iodine concentrations: 37.0, 18.5, 9.25, 4.63, and 0 mg I/mL.

#### Imaging protocols

CT scans were performed with the first-generation clinical PCD-CT (NAEOTOM Alpha, Siemens Healthineers, Erlangen, Germany) with the software version Syngo CT VA40A. For every combination of the silicone tube and iodine concentration, scans with six different fixed tube currents (13, 25, 38, 50, 127, and 380 mAs) were acquired using a tube voltage of 120 kVp, resulting in the CT dose indices (CTDIs) of 1, 2, 3, 4, 10, and 30 mGy, respectively. All images were reconstructed as VMIs at 55 keV in the vascular spectral post-processing format, which is the vendor’s standard keV level for CT angiographies and consistent with the recommendations of a previous study that investigated the effect of different keV levels [26]. The quantitative kernel (Qr) was applied with each available sharpness level, *i.e.*, Qr36, Qr40, Qr44, Qr48, Qr56, Qr60, Qr64, Qr68, Qr72, and Qr76. The choice of the quantitative kernel instead of a vascular kernel was made because the Qr kernels were optimized for further spectral investigations such as plaque composition analysis. Every quantitative kernel was combined with three iterative reconstruction levels of the quantum iterative reconstruction (QIR) algorithm (QIR-2, QIR-3, and QIR-4), resulting in a total number of 30 reconstructions per dose level. A previous study demonstrated the inferiority of filtered back projection reconstructions (QIR-off) and first QIR level (QIR-1) for coronary PCD-CTA [21]. Therefore, they were not analyzed in this study. Images were reconstructed with a slice thickness of 0.4 mm, a 512 × 512 matrix, and a field-of-view of 205 × 205 mm^2^ centered around the silicone tube. This yielded isotropic voxels with an edge length of 0.4 mm.

#### Image analysis

**Fig. 1:**
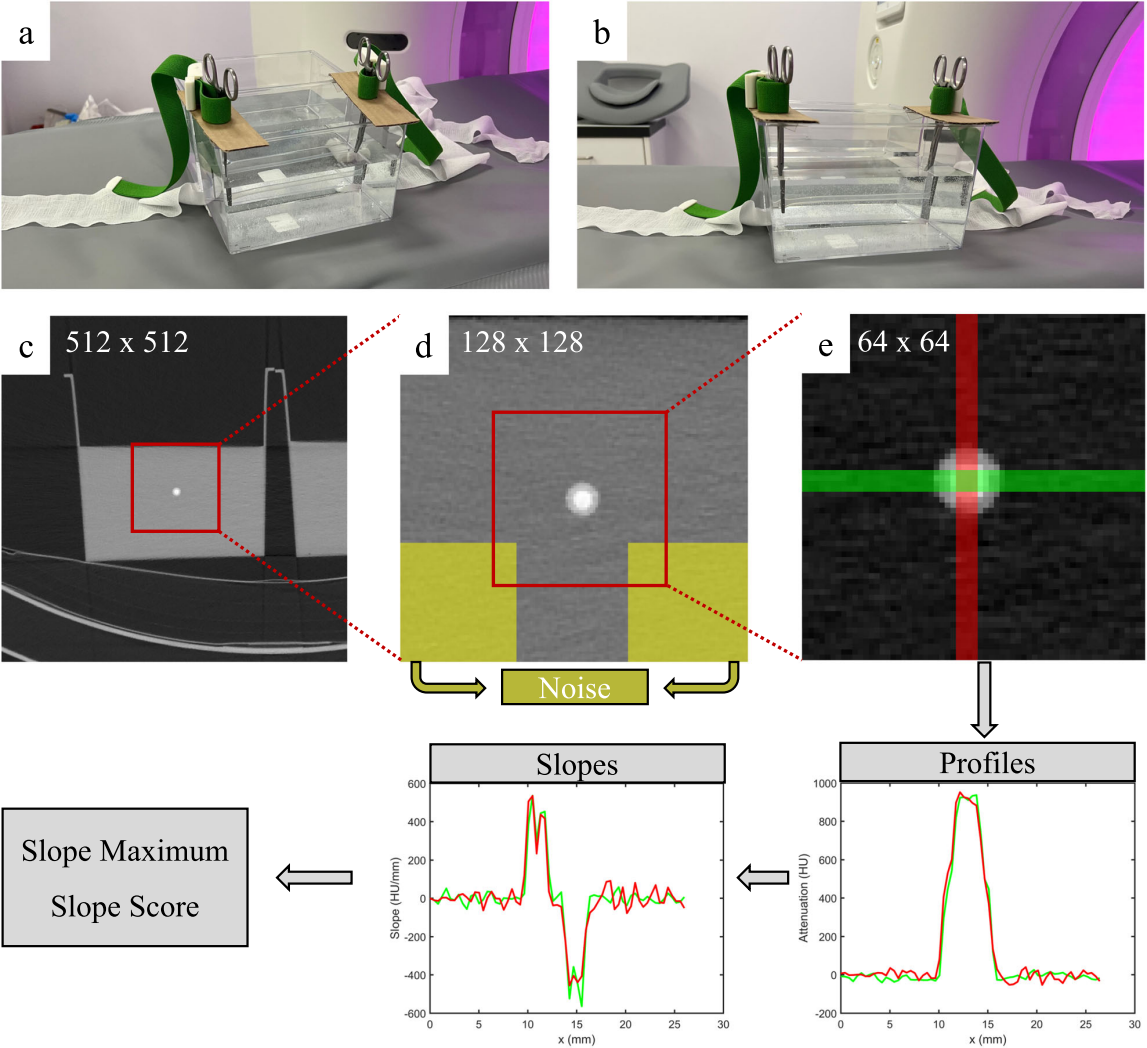
Image analysis scheme. **a**, **b** Photographs of the experimental setup. The initial images (**c**) are cropped centered (**d**) to determine image noise, and once again center cropped (**e**) for the extraction of the edge profiles and the slopes. The example shows the image of the silicone tube with an inner diameter of 3 mm filled with an iodine concentration of 37 mg I/mL. The slope score in this example is 8

Image analyses were performed on the computing platform MATLAB (version R2021b, The MathWorks, Inc., Natick, USA). For each reconstruction, a single slice in the middle of the containers was used. First, these 512 × 512 images were cropped to the 128 × 128 central regions. In these, the noise levels were defined by the standard deviation of the water attenuation in the 44 × 44 regions on the bottom left and bottom right. The images were cropped a second time to a 64 × 64 region centered around the silicone tubes. Horizontal and vertical edge profiles were determined by averaging the attenuation of four neighboring pixels perpendicular to the profile direction. The amplitude of profiles was achieved by the maximum values and averages of the horizontal and vertical profiles. The slope at any point of these profiles was determined by the linear regression of each three neighboring points. The maximal slope values at the edges of the silicone pipe were determined, *i.e.*, at the transition from the water surrounding the wall or from the wall to the inner volume. The mean slope maximum was calculated by the mean of these four maxima. Additionally, a slope score was defined by the count of the local slope maxima, which were visible and above the noise level. This score could reach a maximum of eight when the slope of all four transitions from the surrounding water to the tube wall and of all four transitions from the pipe wall to the inner volume are visible as individual local maxima. Figure 1 illustrates the image analysis scheme.

### *In vivo* study

#### Study population and imaging protocol

The study population consisted of 20 consecutive patients, who received a clinically indicated CTA of the lower extremities on a PCD-CT (NAEOTOM Alpha, Siemens) with the software version syngo CT VA40A SP1 in April and May 2023. Images were acquired with a tube voltage of 120 kVp. Tube current modulation (CARE Dose4D, Siemens) was used with the image quality index set to 145. All images were reconstructed as VMIs at 55 keV with 0.4 mm isotropic voxels using a medium (Qr44), a sharp (Qr60), and an ultrasharp (Qr72) kernel. For each kernel, three different iterative reconstruction levels were applied (QIR-2, QIR-3, and QIR-4). Contrast media injection protocols consisted of a single bolus volume of 80 mL, with a flow of 4 mL/s and iodine flux of 1.5 gI/s (Ultravist® 370, Bayer Vital, Leverkusen, Germany) followed by a saline bolus volume of 40 mL, with a flow of 3 mL/s. Acquisitions were triggered by bolus tracking in the proximal abdominal aorta with a signal threshold of 100 HU.

#### Qualitative image analysis

Three readers rated the image quality—one board-certified consultant radiologist with 15 years and two residents with 2 and 4 years of experience in the field. For every patient, all nine reconstructed images (three kernels with three QIR levels, respectively) were arranged side-by-side in a random order using the institutional Picture Archiving and Communication System−PACS (Sectra®, Linköping, Sweden). All images were anonymized and blinded for reconstruction parameters. Standard angiography window settings were used (width 1,100, level 300) with the possibility to change the windowing settings separately for each image. Consistent with previous studies [22, 24, 28], a 5-point Likert scale was used for the assessment of the following criteria: overall image quality (1: very poor/non-diagnostic, 2: poor, 3: moderate, 4: good, 5: excellent), image noise (1: very strong, 2: strong, 3: moderate, 4: little, 5: no/very little), and delineation of plaques and vessel walls (1: unacceptable/non-diagnostic, 2: suboptimal, 3: acceptable, 4: good, 5: precise).

### Statistical analysis

All statistical analyses were conducted using dedicated statistical software (R, version 4.1.1, R Foundation for Statistical Computing, Vienna, Austria). Categorical and binary baseline parameters were presented as absolute numbers and percentages, while ordinal-scaled variables were described using medians and interquartile ranges. For interval-scaled variables that followed a normal distribution according to the Shapiro–Wilk test, means and standard deviations were reported. To determine statistical differences in quantitative and qualitative image parameters, paired samples Wilcoxon rank tests with Bonferroni correction for multiple comparisons were used. A *p*-value < 0.050 was considered statistically significant.

Inter-reader agreement was assessed using Krippendorff’s *α* coefficient with the following interpretation: poor agreement for values in the range of 0.0 to 0.2, fair agreement for values greater than 0.2 up to 0.4, moderate agreement for values greater than 0.4 up to 0.6, substantial agreement for values greater than 0.6 up to 0.8, and excellent (or near-perfect) agreement for values greater than 0.8 up to 1.0 [28].

## Results

### Phantom study

**Fig. 2:**
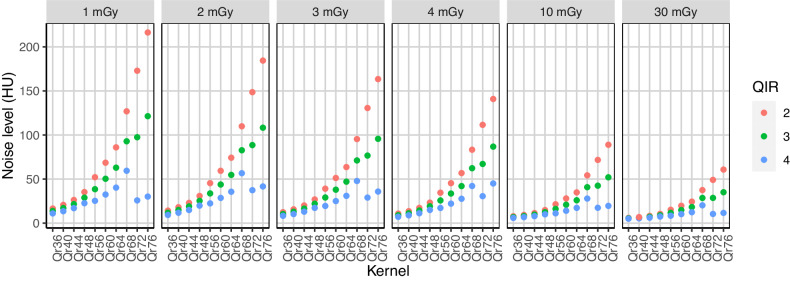
Dependence of image noise on the sharpness of the reconstruction kernel, the quantum iterative reconstruction (QIR) level, and the applied radiation dose. Noise levels are shown for the scans of the silicone tube with an inner diameter of 1 mm filled with an iodine concentration of 37 mg I/mL

Noise levels for the different kernels, CTDIs, and QIR levels are shown in Fig. 2. Noise levels clearly increased when increasing kernel sharpness and keeping the radiation dose constant (*e.g.*, at 3 mGy and using a QIR level of 3: noise level of 16 HU for Qr44, 38 HU for Qr60, and 77 HU for Qr72, *p* < 0.001). Image noise showed an inverse relationship with increasing levels of QIR (*e.g.*, at 3 mGy and reconstructed with the Qr60 kernel: noise level of 51 HU at QIR-2 *versus* 38 HU at QIR-3 *versus* 25 HU at QIR-4, *p* < 0.001). The provided noise levels refer to the images with a 1 mm silicone tube filled with a 37 mg I/mL iodine solution. However, the tube diameter and the iodine concentration showed no relevant influence on the noise levels (*p* > 0.999).

**Fig. 3:**
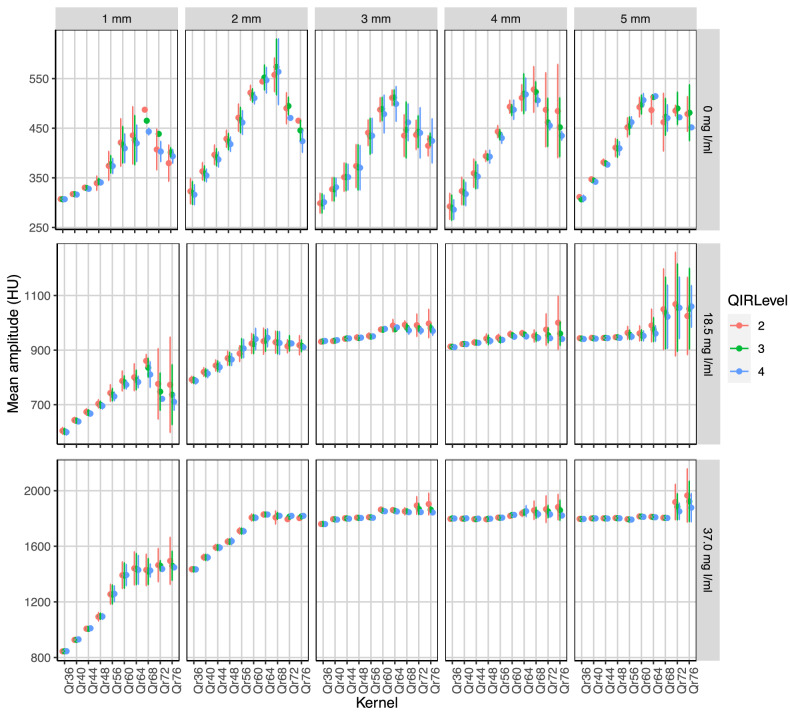
Dependency of the amplitude on the reconstruction kernel and the quantum iterative reconstruction (QIR) levels. Results are shown for the tube with five different inner diameters and three different iodine concentrations scanned with a CTDI of 3 mGy. The results for the concentration of 0 mg I/mL depict the amplitude of the tube wall. Dependency on the radiation dose is provided in the supplementary material

The dependency of the mean amplitude of the attenuation profile on kernel sharpness in the iodine-filled tubes is shown in Fig. 3. In the tubes with a diameter ≤ 2 mm, the mean amplitude is reduced in the images reconstructed with a soft kernel (*e.g.*, at 3 mGy, 18.5 mg I/mL iodine solution, QIR-4, and 2 mm diameter: Qr36, 787 ± 12 HU; Qr44, 838 ± 18 HU; Qr60, 940 ± 39 HU; *p* ≤ 0.003). This effect was not observed for the kernels with a sharpness level ≥ 60 (*p* > 0.999). The results for the tubes filled with saline (0 mg I/mL) depict the mean amplitude of the silicone wall. Similar to the iodine-filled tubes, the mean wall amplitude was significantly reduced for kernel sharpness levels below 60 (*e.g.*, at 3 mGy, QIR-4 and 2 mm diameter: Qr36, 316 ± 20 HU; Qr44, 387 ± 15 HU; Qr60, 511 ± 12 HU; *p* < 0.001).

**Fig. 4:**
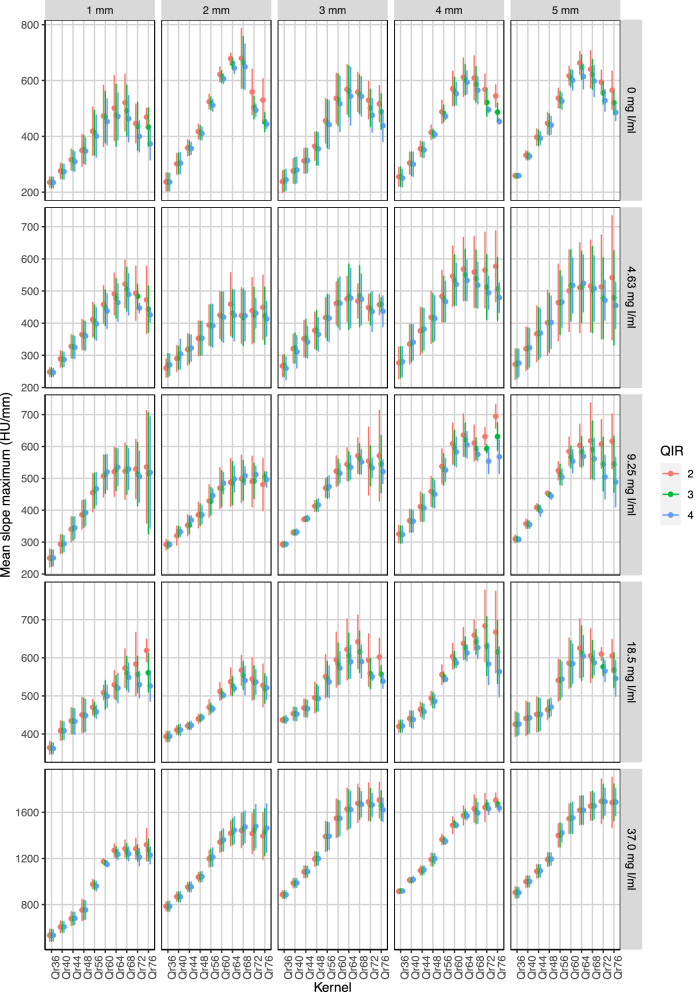
Dependency of the slope maximum on the reconstruction kernel for the different inner diameters and iodine concentrations. Results are shown for the tubes scanned with a CTDI of 3 mGy. The results of the other radiation doses are provided in the Supplementary material

Increased sharpness level of the kernel yielded higher slope maxima at the edges of the silicone tube—both at the outer edge to the saline surrounding and at the inner edge to the lumen (Fig. 4). While sharpness levels between 36 and 56 showed maximum slopes reduced by 16% to 47% of the maximum at sharpness level 76, maximum slopes of all kernels with sharpness level of 60 or higher differed by less than 10% (see Table 1). QIR had no significant influence on the maximum slope (*p* > 0.999).

**Table 1:** Characteristics of the modulation transfer functions (MTF) and maximum slopes for the 5-mm tube filled with 37 mg I/mL and scanned with a CTDI of 3 mGy and QIR-4

Kernel	ρ_50_ (Lp/cm)	ρ_10_ (Lp/cm)	Maximum slope (HU/mm)	Relative difference to Qr76
Qr36	3.4	5.6	900 ± 50	-47%
Qr40	4.0	6.6	1,000 ± 50	-41%
Qr44	4.6	7.7	1,090 ± 50	-35%
Qr48	5.4	9.0	1,200 ± 50	-29%
Qr56	7.4	10.8	1,420 ± 80	-16%
Qr60	8.8	11.2	1,550 ± 140	-9%
Qr64	10.1	11.2	1,620 ± 130	-4%
Qr68	11.8	13.9	1,660 ± 110	-2%
Qr72	14.1	17.3	1,690 ± 120	0%
Qr76	15.8	18.7	1,690 ± 120	0%

*ρ*_50_ and *ρ*_10_ represent the spatial frequencies at which the MTF decreases to 50% and 10% of its value at *ρ* = 0 Lp/cm. Mean data with a standard deviation of the maximum slope is presented. *Lp* Line pairs

The results of the slope scores, which evaluate the slope maxima in relation to the noise level, are shown in the Supplementary Material (Figs. S1 to S5). In the images scanned with a CTDI of 3 mGy, typical CTDI at the BTK level, iodine yielded higher attenuation than the vessel wall, therefore, soft kernels could not separate the two slope maxima at the tube wall. Kernels with higher sharpness levels overcame this restriction. However, caused by the increased noise level, ultrasharp kernels reached lower scores in several cases. Higher QIR levels improved the slope scores. These effects were even more pronounced in the images scanned with lower CTDIs. In contrast, higher CTDIs could partially overcome the limitations of ultrasharp kernels by the reduction of noise levels. In all acquired images with an iodine concentration of 9.25 mg I/mL (not shown in the figures), slope scores reached only a value of 4, since the attenuation difference of the silicone wall and iodine was too small to separate a local slope maximum.

### *In vivo* study

The baseline characteristics of the 20 patients scanned in this study are listed in Table 2. The median effective CTDI in the lower leg area was 2.51 mGy (2.50–2.57).

**Table 2:** Baseline characteristics (*n* = 20)

Age (years)	67 (63–80)	
Male sex (*n*)	16 (80%)	
Body height (cm)	174.5 (170–178)	
Body weight (kg)	78.0 (73.5–85.8)	
Body Mass Index (kg/m^2^)	25.6 (24.2–28.1)	
Patients with peripheral artery disease (*n*)	18	
Fontaine stage (*n*)		
IIa	1	
IIb	8	
III	3	
IV	6	
Patients with plaques in the evaluated regions (*n*)	15	
Patients with (*n*)	Popliteal	BTK
Calcified plaques	9	11
Noncalcified plaques	5	8
Mixed plaques	9	7

Median data is shown with interquartile ranges in parentheses. *BTK* Below-the-knee

**Fig. 5:**
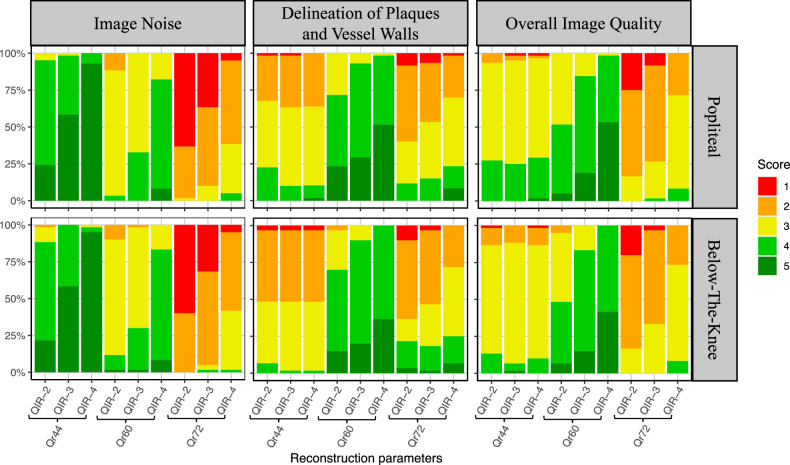
Qualitative image analyses of image noise, delineation of plaques and vessel walls, and overall CTA image quality at the popliteal (Pop) and below-the-knee (BTK) levels

Table 3 and Fig. 5 showcase the outcome of the qualitative image analysis. No relevant differences were seen between the two evaluated levels (popliteal and BTK), even though the small difference in the delineation reached statistical significance (*p* = 0.040). All three readers found increasing noise levels (lower scores) with an increase in the kernel sharpness. For all three kernels, an effective reduction of noise was seen by the increase of the QIR level (*p* < 0.050). By the increase of the QIR level from 2 to 4, the image noise changed from little (score 4) to very little (score 5) for the medium kernel (Qr44), from moderate (score 3) to little (score 4) for the sharp kernel (Qr60), and from very strong (score 1) to strong (score 2) for the ultrasharp kernel (Qr72).

**Table 3:** Median scores of qualitative image analyses

Reconstruction		Image noise		Delineation of plaques and vessel walls		Overall image quality	
		Popliteal	BTK	Popliteal	BTK	Popliteal	BTK
Qr44	QIR-2	4 (4–4)	4 (4–4)	3 (2–3)	2 (2–3)	3 (3–4)	3 (3–3)
	QIR-3	5 (4–5)	5 (4–5)	3 (2–3)	2 (2–3)	3 (3–3)	3 (3–3)
	QIR-4	5 (5–5)	5 (5–5)	3 (2–3)	2 (2–3)	3 (3–4)	3 (3–3)
Qr60	QIR-2	3 (3–3)	3 (3–3)	4 (3–4)	4 (3–4)	4 (3–4)	3 (3–4)
	QIR-3	3 (3–4)	3 (3–4)	4 (4–5)	4 (4–4)	4 (4–4)	4 (4–4)
	QIR-4	4 (4–4)	4 (4–4)	5 (4–5)	4 (4–5)	5 (4–5)	4 (4–5)
Qr72	QIR-2	1 (1–2)	1 (1–2)	2 (2–3)	2 (2–3)	2 (2–2)	2 (2–2)
	QIR-3	2 (1–2)	2 (1–2)	3 (2–3)	2 (2–3)	2 (2–3)	2 (2–3)
	QIR-4	2 (2–3)	2 (2–3)	3 (2–3)	3 (2–3)	3 (2–3)	3 (2–3)

Interquartile ranges are presented in parentheses. *BTK* Below-the-knee, *Pop* Popliteal

The images reconstructed with the sharp kernel (Qr60) reached the highest scores in the delineation of plaques and vessel walls. The delineation in these images was predominantly classified as good (score 4), whereas in the other images, it was rated suboptimal (score 2) to acceptable (score 3). None of the three readers observed any reduction in delineation caused by the QIR algorithm, *e.g.*, due to blurring effects. In contrast, images with QIR-4 reached even higher scores (*p* < 0.050), probably caused by the reduction of image noise.

The images reconstructed with the sharp kernel reached the best scores in the delineation of the plaques and vessel walls (for the pair-wise comparisons with Qr60 QIR-4 all *p* ≤ 0.001, except to Qr60 QIR-3 with *p* = 0.298 on the popliteal and *p* = 0.307 on the BTK level). The overall image quality of the sharp kernels with the highest QIR level (QIR-4) was rated best with good to excellent scores (for the pair-wise comparisons with Qr60 QIR-4, all *p* ≤ 0.002).

Altogether, inter-rater reliability was substantial (*α* = 0.71 for the popliteal, and 0.70 for the BTK level). Looking at the individual criteria, an excellent agreement was found for image noise (popliteal, *α* = 0.86; BTK, *α* = 0.84), a moderate agreement for delineation of plaques and vessel walls (popliteal, *α* = 0.53; BTK, *α* = 0.52), and a substantial agreement for overall image quality (popliteal, *α* = 0.66; BTK, *α* = 0.68).

**Fig. 6:**
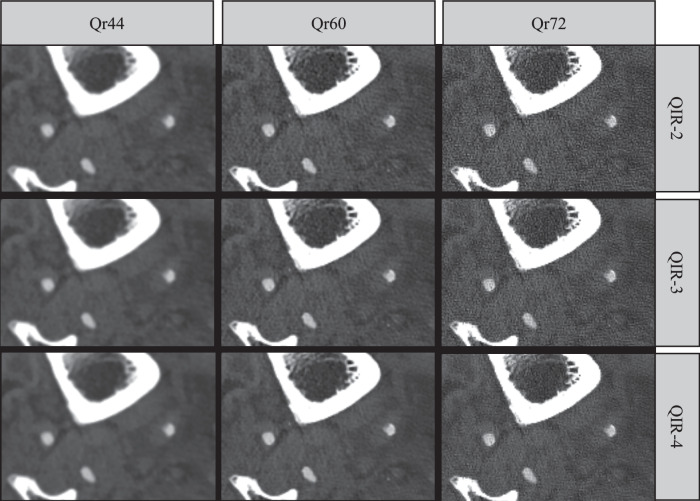
Image example at the below-the-knee level. The example shows a noncalcified plaque of the posterior tibial artery of an 80-year-old male patient who had a wet necrosis of the right heel. The images are reconstructed with the medium (Qr44), sharp (Qr60), and ultrasharp (Qr72) kernel, and with three different levels of the quantum iterative reconstruction algorithm (QIR-2 to QIR-4). Window settings: width 1,500; level 600

**Fig. 7:**
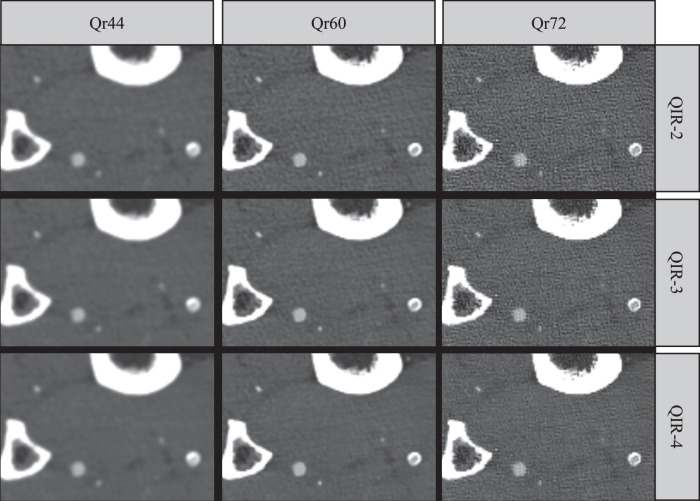
Image example at the below-the-knee level. The example shows a circular calcification of the posterior tibial artery and an occlusion of the anterior tibial artery of a 69-year-old female patient who had Fontaine stage IIb of peripheral artery disease. The images are reconstructed with the medium (Qr44), sharp (Qr60), and ultrasharp (Qr72) kernel, and with three different levels of the quantum iterative reconstruction algorithm (QIR-2 to QIR-4). Window settings: width 1,500; level 500

Image examples reconstructed with the different kernels and QIR levels are shown in Figs. 6 and 7.

## Discussion

In this *in vitro* study with *in vivo* validation, optimal reconstruction parameters for PCD-CTA images of the BTK arteries were defined. A sharp reconstruction kernel (Qr60) in combination with the highest available QIR level (QIR-4) provided the best image quality for arterial plaque analysis without suffering from image blurring or excessive noise levels.

Enhancing the diagnostic accuracy of lower extremity CTA holds significant clinical importance, given its widespread adoption as a standard procedure for assessing patients with peripheral arterial disease [2, 29]. It is increasingly being utilized to guide treatment decisions (surgical *versus* transluminal) and interventional treatment planning [30, 31]. However, the assessment of small BTK vessels with a high prevalence of arteriosclerosis remains challenging [6, 32]. Salimova et al [7] showed an image quality improvement of the BTK level by small field-of-view reconstructions due to a higher in-plane spatial resolution. Additional improvements could be reached by utilizing dual-energy CT with its spectral capabilities such as VMIs or calcium removal [8–10]. Despite these advances, dual-energy CT still exhibits lower diagnostic accuracy when compared to DSA, especially in the BTK arteries.

Two preliminary studies investigated the potential of PCD-CTA in the lower extremities [26, 27]. Both studies used a 2.5-fold higher slice thickness of 1 mm, compared to our study. Rippel et al [26] analyzed the effect of different keV levels of the VMIs reconstructed with manually adapted fields of view with an estimated in-plane resolution between 0.6 × 0.6 and 0.7 × 0.7 mm^2^, Qr36 kernel, and QIR-3. Their study shows that using low-keV VMI reconstructions yields considerably improved contrast-to-noise ratios compared to 80 kVp and 100 kVp EID-CT acquisitions, all while maintaining consistent subjective image quality. Gruschwitz et al [27] analyzed the benefit of PCD-CTA using a cadaveric upper leg model with images reconstructed with a slice thickness of 1 mm and an in-plane resolution of 0.3 × 0.3 mm^2^ using the Bv48 kernel and QIR-3. They demonstrated the potential to achieve up to an 83% reduction in radiation exposure compared to EID-CT, while also offering enhanced luminal contrast attenuation, noise reduction, and improved image quality. However, even more pronounced benefits of PCD-CTA could be expected using the optimized reconstruction parameters identified in our study.

Thus far, spectral reconstructions on the PCD-CT scanner, such as VMIs or material decomposition, are limited to a slice thickness of 0.4 mm or higher. For this reason, the images were reconstructed as 0.4 mm isotropic voxels and with the quantitative kernels (Qr), which are specifically designed for optimizing spectral post-processing, ensuring the provision of highly stable data, particularly for tasks such as plaque composition analysis [33]. This might provide both prognostic imaging biomarkers and predictors for therapy success for BTK plaques, as was shown in previous studies for superficial femoral artery occlusions, *e.g.*, in an MRI study by McDermott et al [34], and in a study by Hartwig et al, who used plaque analysis with optical coherence tomography [35].

In the applied standard acquisition mode, the maximum in-plane spatial resolution, given by the size of the detector elements, is 0.2 mm [36]. Since the used voxel size of 0.4 mm exceeds this value, it determines the maximum possible spatial frequency of the reconstruction. According to the Nyquist theorem, the maximum spatial frequency ρ_max_ is 12.5 Lp/cm. To exhaust the given spatial resolution, the reconstruction kernel must not suppress these high spatial frequencies, *i.e.*, its modulation transfer function should not be significantly reduced in this specific frequency range [22]. Softer kernels than the optimum kernel suppress these spatial frequencies too much and lead to a blurring of the image with reduced delineation of edges at the vessel walls or the plaque borders. This effect could be seen in our results for both the mean amplitude (see Fig. 3) and the mean slope maximum (see Fig. 4), which were reduced for kernels that were too soft. The results of the slope scores (c.f. supplementary material) also show this effect. Especially for higher iodine concentrations, soft kernels lead to a blooming of the inner tube volume, whereby the tube wall is overlaid and the boundary to the surrounding area can no longer be demarcated. This resulted in reduced slope scores for higher iodine concentrations and softer kernels. On the other side, ultrasharp kernels have the potential to transfer even higher spatial frequencies. However, because the voxel size determines the maximum spatial frequency, the utilization of ultrasharp kernels primarily results in amplified image noise without providing substantial improvements in image sharpness. In the case of 0.4 mm isotropic voxels, the ρ_10_ values of the kernels Qr68 to Qr76, *i.e.*, the spatial frequency at which the modulation transfer function value drops to 10% of its value at *ρ* = 0 Lp/cm [22], exceed the maximum spatial frequency ρ_max_ (see Table 1). This mainly explains some nonlinear results observed with these kernel sharpnesses, for example, the decrease of the noise (see Fig. 2) and the decrease of the mean amplitude (see Fig. 3), which is accompanied by a reduction of the mean maximum slope (see Fig. 4).

Waiving spectral capabilities, the PCD-CT scanner allows for higher spatial resolutions using ultrahigh resolution (UHR) mode with a slice thickness down to 0.2 mm. An improvement of the spatial resolution requires an increase in the kernel sharpness to fully exploit these enhancements. Potentially, the usage of dedicated edge-attenuating kernels, like the vascular kernels Bv, might further improve the delineation of vessel walls and plaques. Mergen et al [22] identified the Bv64 kernel to optimize UHR coronary PCD-CTA with non-isotropic voxels with 0.2 mm slice thickness and 0.4 × 0.4 mm^2^ in-plane resolution. However, these results are not directly transferable to lower extremity PCD-CTA, since in coronary PCD-CTA the periodic movement of the heart requires ECG triggering or gating which influences the reconstruction procedure. In addition, the UHR mode with 0.2 mm slice thickness typically requires higher radiation doses to compensate for increased image noise. The advantages in diagnostic certainty and treatment allocation of UHR PCD-CTA must be demonstrated in future studies to justify the applications of higher radiation doses in routine clinical practice. In contrast to the UHR mode, the diagnostic power of the standard resolution mode down to a slice thickness of 0.4 mm could be further improved by more pronounced exploitation of spectral capabilities such as iodine maps or a vascular calcium removal algorithm [37].

In comparison to DSA, Meyer et al [6] reported a reduction of sensitivity and specificity in the grading of steno-occlusive lesions with increased calcification using conventional CTA with a slice thickness of 2 mm. As shown in this study, PCD-CTA with optimized reconstruction parameters yields improved delineation of plaques. Therefore, clearly reduced blooming artifacts are expected with the potential of improved qualitative and quantitative diagnosis overcoming the restrictions of increased calcifications. An alternative modality for the imaging of the BTK and pedal arteries is contrast-enhanced magnetic resonance angiography. Its advantages are the capability to conduct multiphasic imaging comparable to DSA, eliminating exposure to ionizing radiation, and achieving this with a minimal amount of contrast material [38]. However, the spatial resolution of contrast-enhanced magnetic resonance angiography does not reach that of PCD-CTA, since even high-resolution protocols are limited to isotropic voxels with an edge length around 1 mm [39–41]. In addition to the existing advantages of CTA such as rapid acquisition and direct visualization of classified plaques, PCD-CTA provides more technical advantages with spectral imaging and UHR acquisition. However, the clinical benefits of these technical improvements need to be proven in further studies.

This study has a few limitations which merit consideration. First, the study was conducted at a single center and the scope of the *in vivo* part of the study was only to preliminarily validate the phantom results, therefore the patient sample size and the number of evaluated plaques were limited. Second, only the quantitative kernel Qr was used. More edge-attenuating kernels might be beneficial under certain circumstances. In a previous study of coronary PCD-CTA, the vascular kernel Bv was preferred over the Qr kernel [28]. Third, VMIs were exclusively reconstructed at 55 keV, which is consistent with the results of the keV level modification by Rippel et al [26]. However, a recent phantom study for coronary PCD-CTA reports the best image quality for the 40 keV VMI level [21]. Even though 40 keV VMIs might further improve image quality, the results of the study concerning the kernel sharpness and the QIR level are expected not to be influenced relevantly by this change in keV level. Fourth, only the three highest QIR levels were considered and FBP was not used as the reference standard. Fifth, CTA images were assessed without comparison to DSA as an invasive reference.

In conclusion, for isotropic voxels with 0.4-mm edge length, a sharp kernel (Qr60) should be used for PCD-CTA of the lower legs. The highest QIR level provides the most effective dose reduction without quality reduction by image blurring. This optimal choice of kernel sharpness and QIR level is necessary for effectively exploiting the potential of PCD-CT for CTA of the lower legs.

### Supplementary information

**Additional file 1: Supplementary Fig. S1.** Dependency of the amplitude on the reconstruction kernel, the quantum iterative reconstruction (QIR) levels, and the radiation dose. Results are shown for the tube with five different inner diameters and three different iodine concentrations. The results for the concentration of 0 mg I/ml depict the amplitude of the tube wall. **Supplementary Fig. S2.** Dependency of the slope maximum on the reconstruction kernel for the different inner diameters and iodine concentrations. **Supplementary Fig. S3.** Slope scores for a CT dose index of 1 mGy. Results are shown for the different sharpness levels of the reconstruction kernel, the different Quantum iterative reconstruction (QIR) levels, the different inner diameters of the silicone pipes and the different iodine concentrations. **Supplementary Fig. S4.** Slope scores for a CT dose index of 2 mGy. Results are shown for the different sharpness levels of the reconstruction kernel, the different Quantum iterative reconstruction (QIR) levels, the different inner diameters of the silicone pipes and the different iodine concentrations. **Supplementary Fig. S5.** Slope scores for a CT dose index of 3 mGy. Results are shown for the different sharpness levels of the reconstruction kernel, the different Quantum iterative reconstruction (QIR) levels, the different inner diameters of the silicone pipes and the different iodine concentration. **Supplementary Fig. S6.** Slope scores for a CT dose index of 4 mGy. Results are shown for the different sharpness levels of the reconstruction kernel, the different Quantum iterative reconstruction (QIR) levels, the different inner diameters of the silicone pipes and the different iodine concentrations. **Supplementary Fig. S7.** Slope scores for a CT dose index of 10 mGy. Results are shown for the different sharpness levels of the reconstruction kernel, the different Quantum iterative reconstruction (QIR) levels, the different inner diameters of the silicone pipes and the different iodine concentrations. **Supplementary Fig. S8.** Slope scores for a CT dose index of 30 mGy. Results are shown for the different sharpness levels of the reconstruction kernel, the different Quantum iterative reconstruction (QIR) levels, the different inner diameters of the silicone pipes and the different iodine concentrations.

## Data Availability

The datasets used and/or analyzed during the current study are available from the corresponding author upon reasonable request.

## Abbreviations

BTK: Below-the-knee
CT: Computed tomography
CTA: CT angiography
CTDI: CT dose index
DSA: Digital subtraction angiography
EID: Energy-integrating detector
PCD: Photon-counting detector
QIR: Quantum iterative reconstruction
UHR: Ultrahigh resolution
VMI: Virtual monoenergetic image

## References

[1] Met R, Bipat S, Legemate DA et al (2009) Diagnostic performance of computed tomography angiography in peripheral arterial disease: a systematic review and meta-analysis. JAMA 301:415–424. 10.1001/JAMA.301.4.415 19176443 10.1001/JAMA.301.4.415

[2] Baliyan V, Shaqdan K, Hedgire S, Ghoshhajra B (2019) Vascular computed tomography angiography technique and indications. Cardiovasc Diagn Ther 9:S14. 10.21037/CDT.2019.07.04 31559151 10.21037/CDT.2019.07.04 PMC6732113

[3] Shwaiki O, Rashwan B, Fink MA et al (2021) Lower extremity CT angiography in peripheral arterial disease: from the established approach to evolving technical developments. Int J Cardiovasc Imaging 37:3101–3114. 10.1007/S10554-021-02277-1 33997924 10.1007/S10554-021-02277-1

[4] Albrecht T, Foert E, Holtkamp R et al (2007) 16-MDCT angiography of aortoiliac and lower extremity arteries: comparison with digital subtraction angiography. AJR Am J Roentgenol 189:702–711. 10.2214/AJR.07.2333 17715120 10.2214/AJR.07.2333

[5] Heijenbrok-Kal MH, Kock MCJM, Hunink MGM (2007) Lower extremity arterial disease: multidetector CT angiography meta-analysis. Radiology 245:433–439. 10.1148/RADIOL.2451061280 17848679 10.1148/RADIOL.2451061280

[6] Meyer BC, Werncke T, Foert E et al (2010) Do the cardiovascular risk profile and the degree of arterial wall calcification influence the performance of MDCT angiography of lower extremity arteries? Eur Radiol 20:497–505. 10.1007/S00330-009-1555-7 19789885 10.1007/S00330-009-1555-7

[7] Salimova N, Hinrichs JB, Gutberlet M et al (2022) The impact of the field of view (FOV) on image quality in MDCT angiography of the lower extremities. Eur Radiol 32:2875–2882. 10.1007/s00330-021-08391-x 34902060 10.1007/s00330-021-08391-x PMC9038851

[8] Brockmann C, Jochum S, Sadick M et al (2009) Dual-energy CT angiography in peripheral arterial occlusive disease. Cardiovasc Intervent Radiol 32:630–637. 10.1007/S00270-008-9491-5 19130122 10.1007/S00270-008-9491-5

[9] Gruschwitz P, Petritsch B, Schmid A et al (2023) Noise-optimized virtual monoenergetic reconstructions of dual-energy CT angiographies improve assessability of the lower leg arterial segments in peripheral arterial occlusive disease. Radiography (Lond) 29:19–27. 10.1016/J.RADI.2022.09.002 36209641 10.1016/J.RADI.2022.09.002

[10] Kosmala A, Weng AM, Schmid A et al (2022) Dual-energy CT angiography in peripheral arterial occlusive disease: diagnostic accuracy of different image reconstruction approaches. Acad Radiol 29:S59–S68. 10.1016/J.ACRA.2020.10.028 33189548 10.1016/J.ACRA.2020.10.028

[11] Willemink MJ, Persson M, Pourmorteza A et al (2018) Photon-counting CT: technical principles and clinical prospects. Radiology 289:293–312 30179101 10.1148/radiol.2018172656

[12] Flohr T, Petersilka M, Henning A et al (2020) Photon-counting CT review. Phys Med 79:126–136. 10.1016/j.ejmp.2020.10.030 33249223 10.1016/j.ejmp.2020.10.030

[13] Leng S, Bruesewitz M, Tao S et al (2019) Photon-counting detector CT: system design and clinical applications of an emerging technology. Radiographics 39:729–743. 10.1148/rg.2019180115 31059394 10.1148/rg.2019180115 PMC6542627

[14] Gutjahr R, Halaweish AF, Yu Z et al (2016) Human imaging with photon counting-based computed tomography at clinical dose levels: contrast-to-noise ratio and cadaver studies. Invest Radiol 51:421–429. 10.1097/RLI.0000000000000251 26818529 10.1097/RLI.0000000000000251 PMC4899181

[15] Rajagopal JR, Farhadi F, Solomon J et al (2021) Comparison of low dose performance of photon-counting and energy integrating CT. Acad Radiol 28:1754–1760. 10.1016/J.ACRA.2020.07.033 32855051 10.1016/J.ACRA.2020.07.033 PMC7902731

[16] Yu Z, Leng S, Kappler S et al (2016) Noise performance of low-dose CT: comparison between an energy integrating detector and a photon counting detector using a whole-body research photon counting CT scanner. J Med Imaging 3:043503. 10.1117/1.JMI.3.4.043503 10.1117/1.JMI.3.4.043503 PMC5155128 28018936

[17] Symons R, Reich DS, Bagheri M et al (2018) Photon-counting CT for vascular imaging of the head and neck: first *in vivo* human results. Invest Radiol 53:135. 10.1097/RLI.0000000000000418 28926370 10.1097/RLI.0000000000000418 PMC5792306

[18] Sartoretti T, Wildberger JE, Flohr T, Alkadhi H (2023) Photon-counting detector CT: early clinical experience review. Br J Radiol. 10.1259/BJR.20220544 10.1259/bjr.20220544 PMC10321251 36744809

[19] Graafen D, Müller L, Halfmann M et al (2022) Photon-counting detector CT improves quality of arterial phase abdominal scans: a head-to-head comparison with energy-integrating CT. Eur J Radiol 156. 10.1016/J.EJRAD.2022.110514 10.1016/j.ejrad.2022.110514 36108479

[20] Graafen D, Emrich T, Halfmann MC et al (2022) Dose reduction and image quality in photon-counting detector high-resolution computed tomography of the chest: routine clinical data. J Thorac Imaging 37:315–322. 10.1097/RTI.0000000000000661 35699680 10.1097/RTI.0000000000000661

[21] Sartoretti T, McDermott M, Mergen V et al (2023) Photon-counting detector coronary CT angiography: impact of virtual monoenergetic imaging and iterative reconstruction on image quality. Br J Radiol 96. 10.1259/BJR.20220466 10.1259/bjr.20220466 PMC9975359 36633005

[22] Mergen V, Sartoretti T, Baer-Beck M et al (2022) Ultra-high-resolution coronary CT angiography with photon-counting detector CT: feasibility and image characterization. Invest Radiol 57:780–788. 10.1097/RLI.0000000000000897 35640019 10.1097/RLI.0000000000000897 PMC10184822

[23] Soschynski M, Hagen F, Baumann S et al (2022) High temporal resolution dual-source photon-counting CT for coronary artery disease: initial multicenter clinical experience. J Clin Med 11. 10.3390/JCM11206003 10.3390/jcm11206003 PMC9604695 36294324

[24] Si-Mohamed SA, Boccalini S, Lacombe H et al (2022) Coronary CT angiography with photon-counting CT: first-in-human results. Radiology 303:303–313. 10.1148/RADIOL.211780 35166583 10.1148/RADIOL.211780

[25] Decker JA, O’Doherty J, Schoepf UJ et al (2023) Stent imaging on a clinical dual-source photon-counting detector CT system-impact of luminal attenuation and sharp kernels on lumen visibility. Eur Radiol 33. 10.1007/S00330-022-09283-4 10.1007/s00330-022-09283-4 36462045

[26] Rippel K, Decker JA, Wudy R et al (2023) Evaluation of run-off computed tomography angiography on a first-generation photon-counting detector CT scanner— comparison with low-kVp energy-integrating CT. Eur J Radiol 158. 10.1016/J.EJRAD.2022.110645 10.1016/j.ejrad.2022.110645 36525704

[27] Gruschwitz P, Hartung V, Kleefeldt F et al (2023) Photon-counting *versus* energy-integrating detector ct angiography of the lower extremity in a human cadaveric model with continuous extracorporeal perfusion. Invest Radiol. 10.1097/RLI.0000000000000982 10.1097/RLI.0000000000000982 37185253

[28] Yang Y, Fink N, Emrich T et al (2023) Optimization of kernel type and sharpness level improves objective and subjective image quality for high-pitch photon counting coronary CT angiography. Diagnostics 13:1937. 10.3390/diagnostics13111937 37296789 10.3390/diagnostics13111937 PMC10252999

[29] Albrecht T, Meyer BC (2007) MDCT angiography of peripheral arteries: technical considerations and impact on patient management. Eur Radiol 17:5–15. 10.1007/S10406-007-0223-8/METRICS 10.1007/S10406-007-0223-8/METRICS 18376452

[30] Fleischmann D, Lammer J (2006) Peripheral CT angiography for interventional treatment planning. Eur Radiol 16:M58–M64. 10.1007/S10406-006-0197-Y/METRICS 18655268 10.1007/S10406-006-0197-Y/METRICS

[31] Itoga NK, Kim T, Sailer AM et al (2017) Lower extremity computed tomography angiography can help predict technical success of endovascular revascularization in the superficial femoral and popliteal artery. J Vasc Surg 66:835–843.e1. 10.1016/j.jvs.2017.02.031 28502550 10.1016/j.jvs.2017.02.031 PMC5898443

[32] Al-rudaini HEA, Han P, Liang H (2018) Comparison between computed tomography angiography and digital subtraction angiography in critical lower limb ischemia. Curr Med Imaging Rev 15:496–503. 10.2174/1573405614666181026112532 10.2174/1573405614666181026112532 32008557

[33] Mergen V, Eberhard M, Manka R et al (2022) First in-human quantitative plaque characterization with ultra-high resolution coronary photon-counting CT angiography. Front Cardiovasc Med 9. 10.3389/FCVM.2022.981012 10.3389/fcvm.2022.981012 PMC9485480 36148053

[34] McDermott MM, Kramer CM, Tian L et al (2017) Plaque composition in the proximal superficial femoral artery and peripheral artery disease events. JACC Cardiovasc Imaging 10:1003–1012. 10.1016/J.JCMG.2016.08.012 27838307 10.1016/J.JCMG.2016.08.012 PMC5701810

[35] Hartwig JW, Braet DJ, Smith JB et al (2021) Optical coherence tomography and plaque morphology for revascularization of the superficial femoral artery. Quant Imaging Med Surg 11:290–299. 10.21037/QIMS-20-707 33392029 10.21037/QIMS-20-707 PMC7719919

[36] Rajendran K, Petersilka M, Henning A et al (2022) First clinical photon-counting detector CT system: technical evaluation. Radiology 303:130. 10.1148/RADIOL.212579 34904876 10.1148/RADIOL.212579 PMC8940675

[37] Allmendinger T, Nowak T, Flohr T et al (2022) Photon-counting detector CT-based vascular calcium removal algorithm. Invest Radiol 57:399–405. 10.1097/RLI.0000000000000853 35025834 10.1097/RLI.0000000000000853 PMC9071027

[38] Primrose CW, Hecht EM, Roditi G et al (2021) MR angiography series: fundamentals of contrast-enhanced MR angiography 41:E138–E139. 10.1148/RG.2021200215 10.1148/RG.2021200215 34197248

[39] Sharafuddin MJ, Stolpen AH, Sun S et al (2002) High-resolution multiphase contrast-enhanced three-dimensional MR angiography compared with two-dimensional time-of-flight MR angiography for the identification of pedal vessels. J Vasc Interv Radiol 13:695–702. 10.1016/S1051-0443(07)61846-6 12119328 10.1016/S1051-0443(07)61846-6

[40] Mohrs OK, Petersen SE, Heidt MC et al (2011) High-resolution 3D non-contrast-enhanced, ECG-gated, multi-step MR angiography of the lower extremities: comparison with contrast-enhanced MR angiography. Eur Radiol 21:434–442. 10.1007/S00330-010-1932-2/FIGURES/4 20706840 10.1007/S00330-010-1932-2/FIGURES/4

[41] Nael K, Krishnam M, Nael A et al (2008) Peripheral contrast-enhanced MR angiography at 3.0T, improved spatial resolution and low dose contrast: initial clinical experience. Eur Radiol 18:2893–2900. 10.1007/S00330-008-1074-Y/TABLES/4 18618122 10.1007/S00330-008-1074-Y/TABLES/4

