# Supplementary material for: In vitro and in vivo optimized reconstruction for low-keV virtual monoenergetic photon-counting detector CT angiography of lower legs
Source: Eur Radiol Exp. 2024 Aug 1;8:89. doi: 10.1186/s41747-024-00481-x (PMC11294310; doi:10.1186/s41747-024-00481-x)
Supplement: Supplementary file 1 — Additional file 1: Supplementary Fig. S1. Dependency of the amplitude on the reconstruction kernel, the quantum iterative reconstruction (QIR) levels, and the radiation dose. Results are shown for the tube with five different inner diameters and three different iodine concentrations. The results for the concentration of 0 mg I/ml depict the amplitude of the tube wall. Supplementary Fig. S2. Dependency of the slope maximum on the reconstruction kernel for the different inner diameters and iodine concentrations. Supplementary Fig. S3. Slope scores for a CT dose index of 1 mGy. Results are shown for the different sharpness levels of the reconstruction kernel, the different Quantum iterative reconstruction (QIR) levels, the different inner diameters of the silicone pipes and the different iodine concentrations. Supplementary Fig. S4. Slope scores for a CT dose index of 2 mGy. Results are shown for the different sharpness levels of the reconstruction kernel, the different Quantum iterative reconstruction (QIR) levels, the different inner diameters of the silicone pipes and the different iodine concentrations. Supplementary Fig. S5. Slope scores for a CT dose index of 3 mGy. Results are shown for the different sharpness levels of the reconstruction kernel, the different Quantum iterative reconstruction (QIR) levels, the different inner diameters of the silicone pipes and the different iodine concentration. Supplementary Fig. S6. Slope scores for a CT dose index of 4 mGy. Results are shown for the different sharpness levels of the reconstruction kernel, the different Quantum iterative reconstruction (QIR) levels, the different inner diameters of the silicone pipes and the different iodine concentrations. Supplementary Fig. S7. Slope scores for a CT dose index of 10 mGy. Results are shown for the different sharpness levels of the reconstruction kernel, the different Quantum iterative reconstruction (QIR) levels, the different inner diameters of the silicone pipes and the di [file 41747_2024_481_MOESM1_ESM.pdf]

In vitro and in vivo optimized reconstruction for low-keV virtual monoenergetic photon-counting detector CT angiography from popliteal to below-the-knee

ELECTRONIC SUPPLEMENTARY MATERIAL

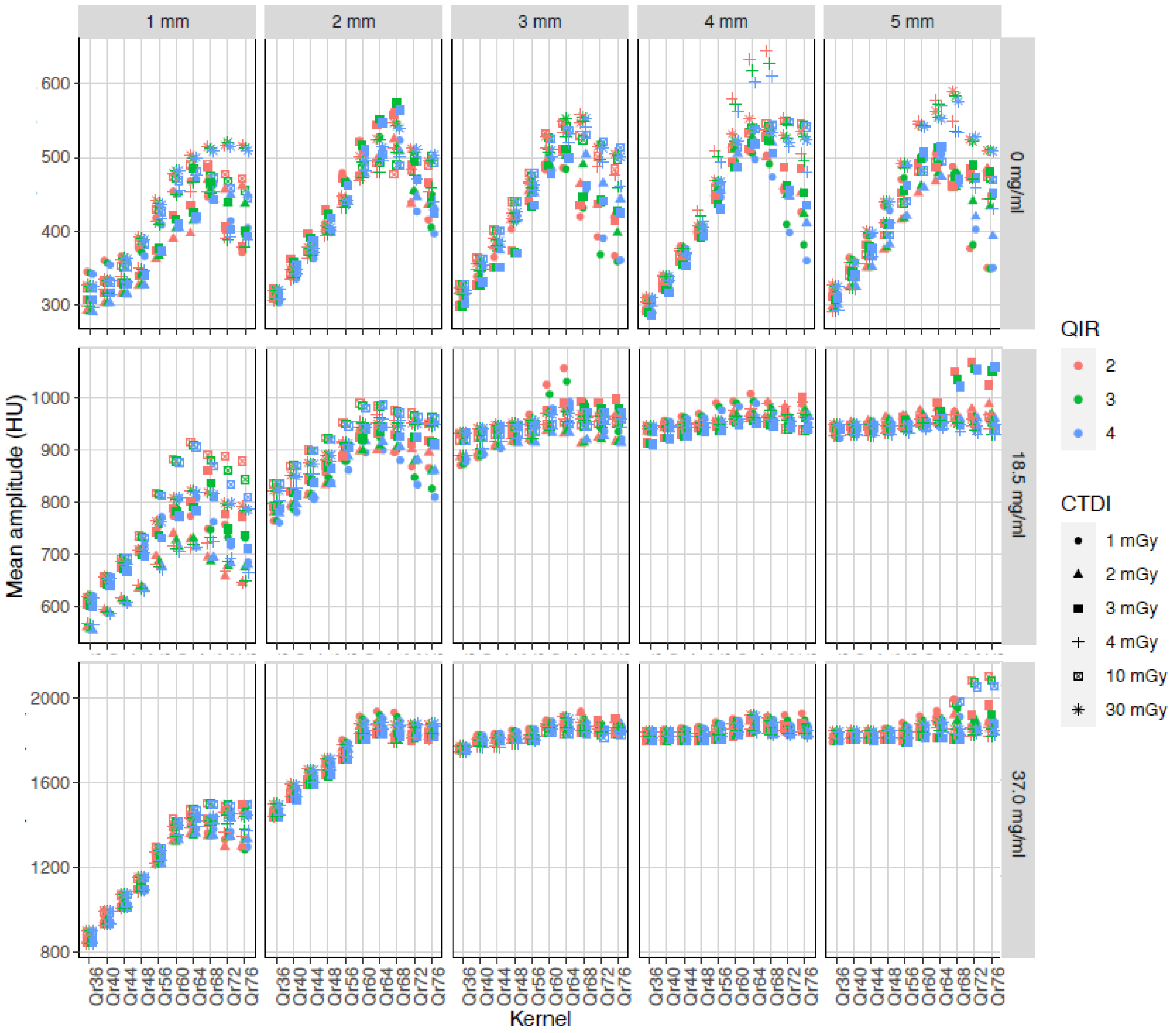

Fig. S1 Dependency of the amplitude on the reconstruction kernel, the quantum iterative reconstruction (QIR) levels, and the radiation dose. Results are shown for the tube with five different inner diameters and three different iodine concentrations. The results for the concentration of 0 mg I/ml depict the amplitude of the tube wall.

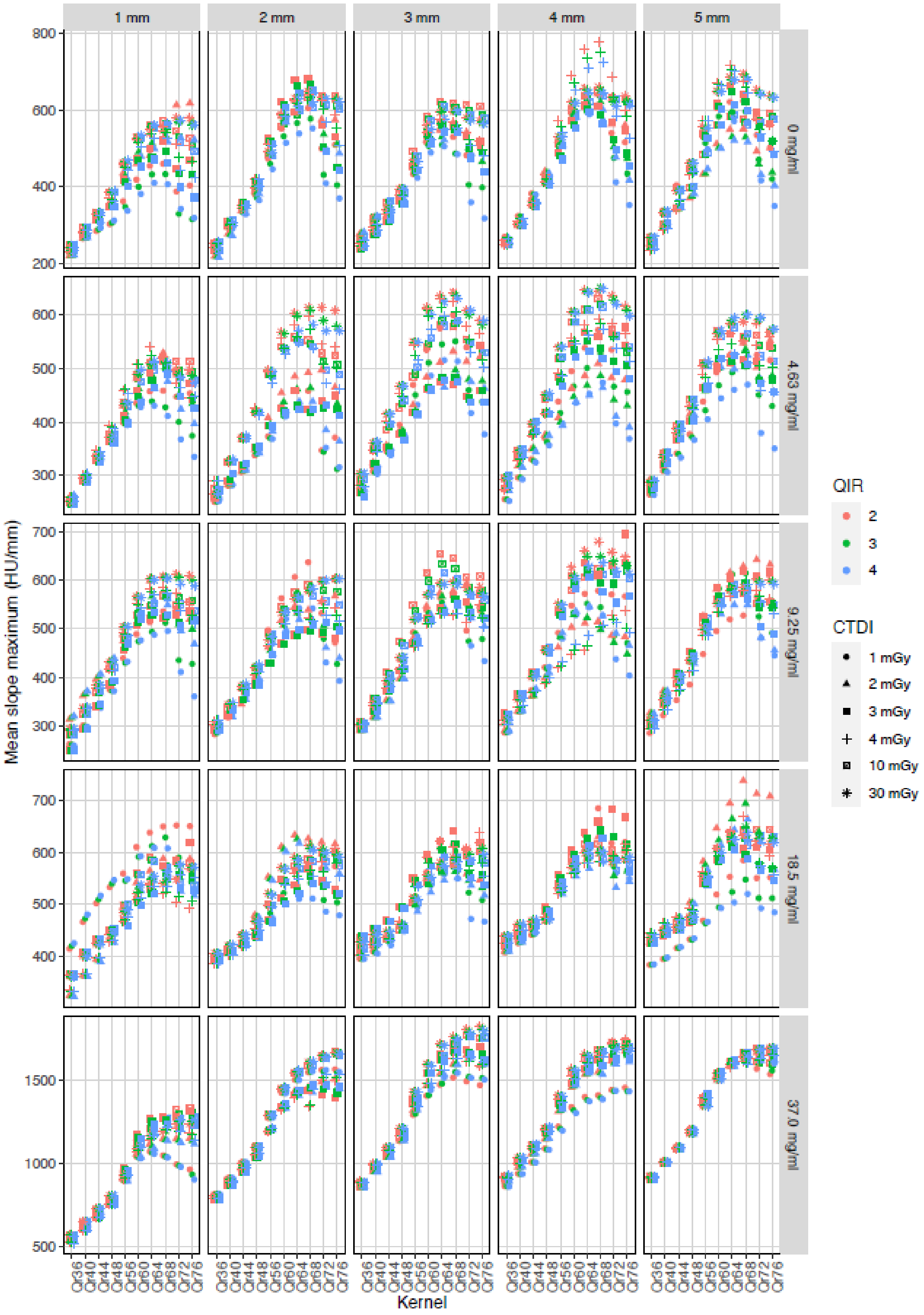

Fig. S2 Dependency of the slope maximum on the reconstruction kernel for the different inner diameters and iodine concentrations.

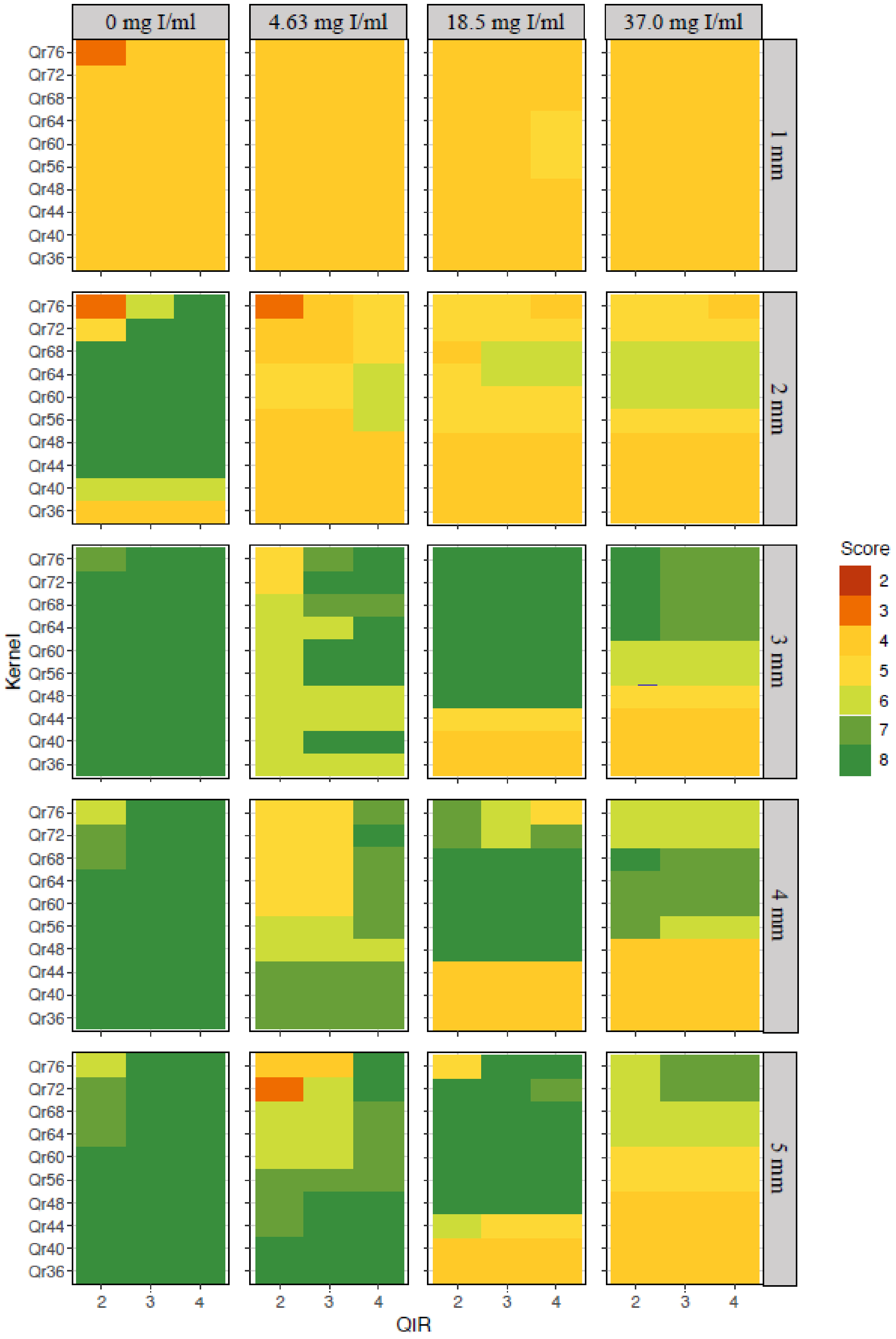

Fig. S3 Slope scores for a CT dose index of 1 mGy. Results are shown for the different sharpness levels of the reconstruction kernel, the different Quantum iterative reconstruction (QIR) levels, the different inner diameters of the silicone pipes and the different iodine concentrations.

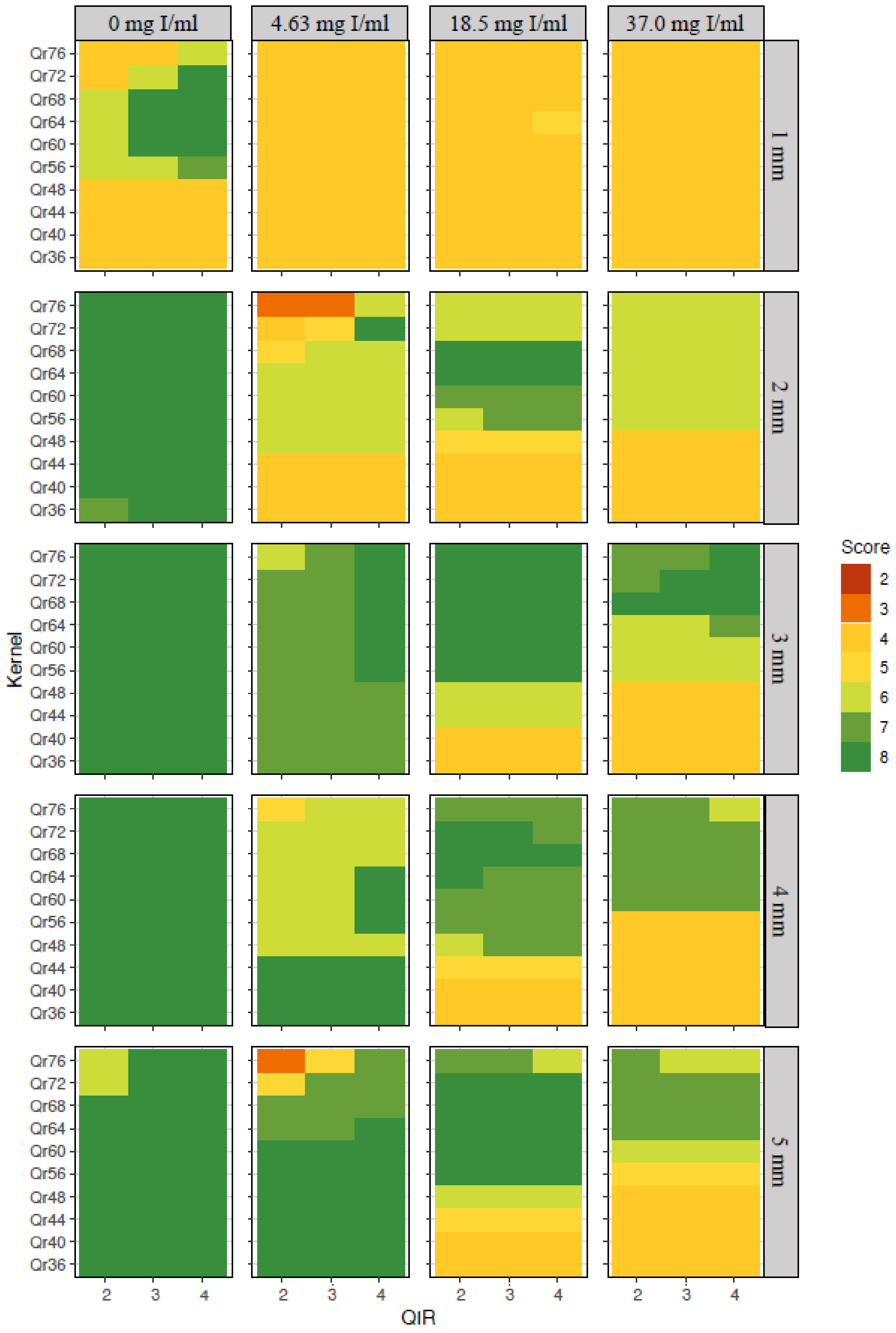

Fig. S4 Slope scores for a CT dose index of 2 mGy. Results are shown for the different sharpness levels of the reconstruction kernel, the different Quantum iterative reconstruction (QIR) levels, the different inner diameters of the silicone pipes and the different iodine concentrations.

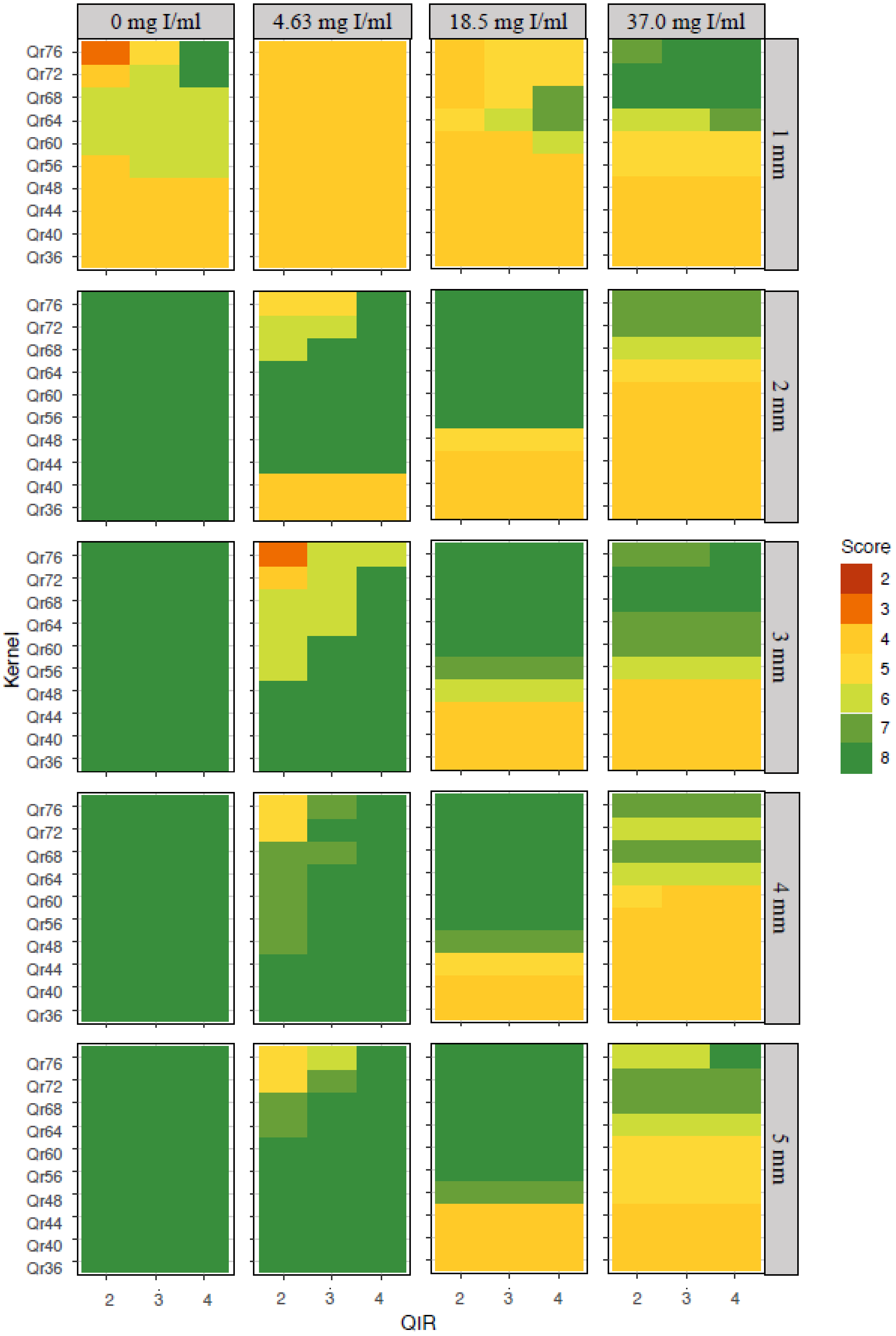

Fig. S5 Slope scores for a CT dose index of 3 mGy. Results are shown for the different sharpness levels of the reconstruction kernel, the different Quantum iterative reconstruction (QIR) levels, the different inner diameters of the silicone pipes and the different iodine concentrations.

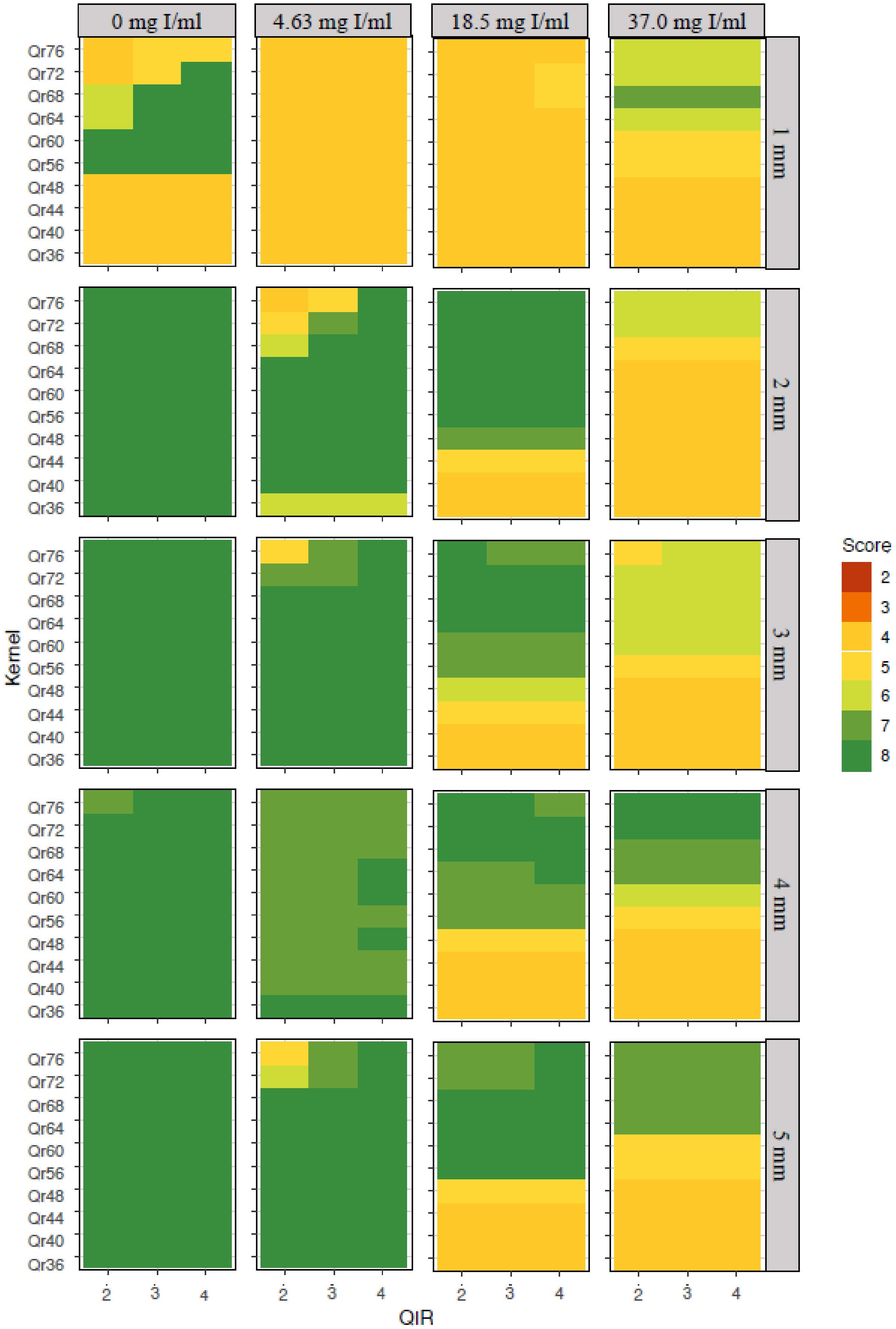

Fig. S6 Slope scores for a CT dose index of 4 mGy. Results are shown for the different sharpness levels of the reconstruction kernel, the different Quantum iterative reconstruction (QIR) levels, the different inner diameters of the silicone pipes and the different iodine concentrations.

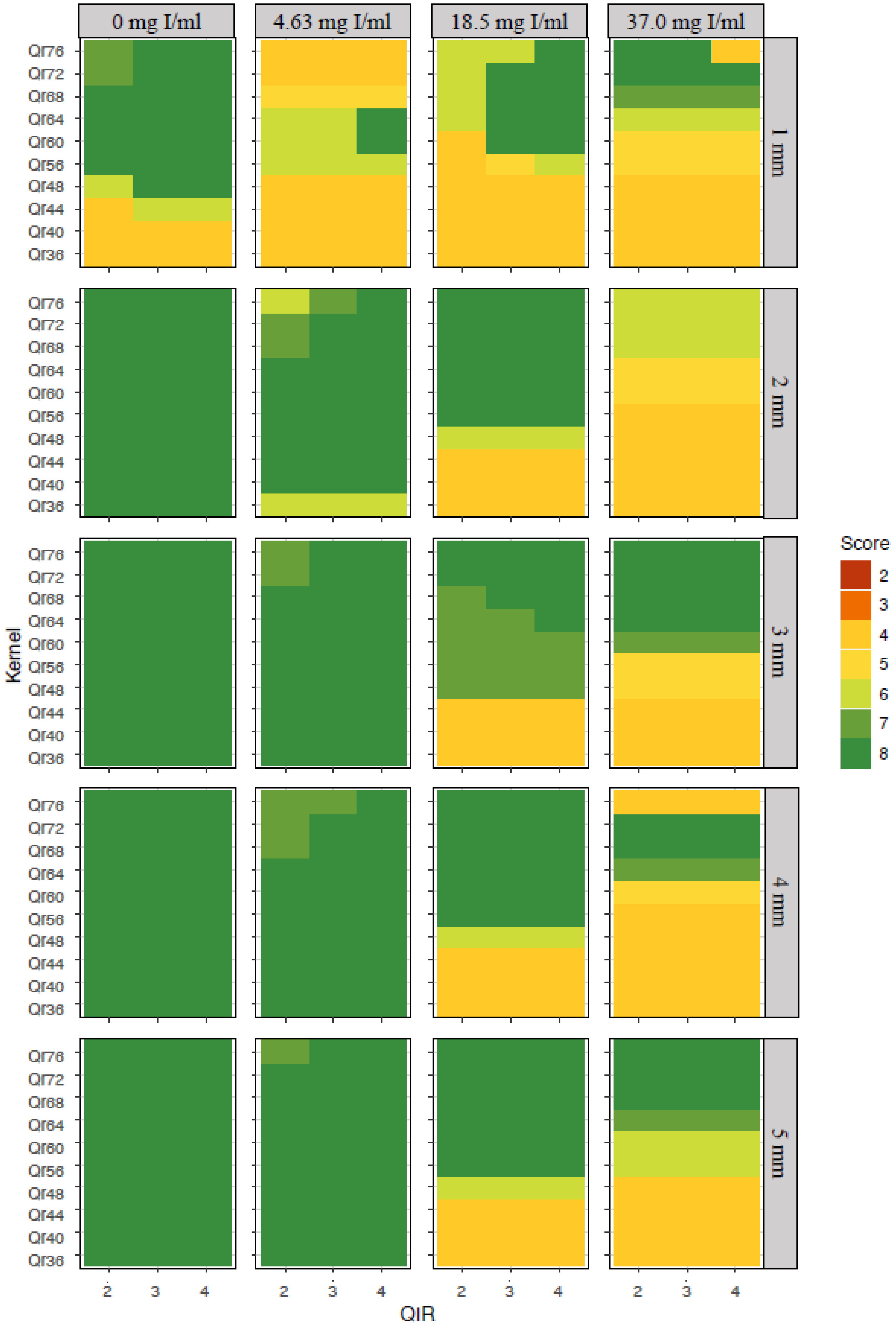

Fig. S7 Slope scores for a CT dose index of 10 mGy. Results are shown for the different sharpness levels of the reconstruction kernel, the different Quantum iterative reconstruction (QIR) levels, the different inner diameters of the silicone pipes and the different iodine concentrations.

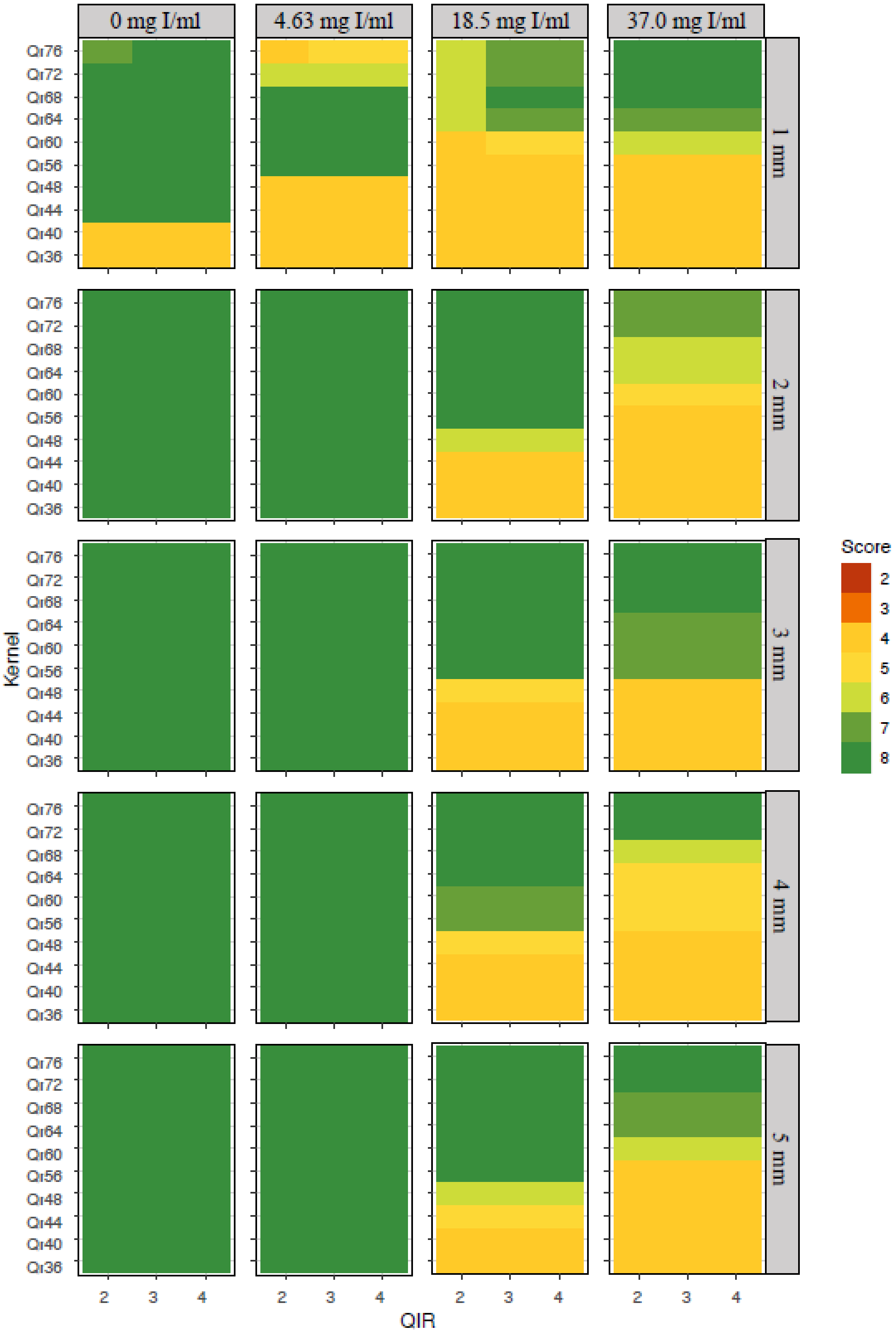

Fig. S8 Slope scores for a CT dose index of 30 mGy. Results are shown for the different sharpness levels of the reconstruction kernel, the different Quantum iterative reconstruction (QIR) levels, the different inner diameters of the silicone pipes and the different iodine concentrations.
